# Central and Peripheral People in a Social Representation Network

**DOI:** 10.5334/irsp.1018

**Published:** 2025-12-04

**Authors:** Pascal Moliner, Graham Dixon

**Affiliations:** 1 Univresité Paul Valéry, Montpellier 3, FR; 2 Ohio State University, USA

**Keywords:** social representation, opinion, network, central, peripheral

## Abstract

Within the framework of network analysis of social representations, attention is directed toward individuals’ centrality within an opinion network. The objective is to identify the characteristics of those who occupy central positions, as well as those on the periphery. To illustrate this approach, we present a study conducted with 1,000 American voters affiliated with the Republican Party, who were surveyed regarding five climate change adaptation policies. The findings indicate that central individuals in the studied network provide more accurate estimations of perceived consensus within the group. Interestingly, the most peripheral individuals appear to form an active minority, engaging in a higher frequency of social interactions within the group. These results suggest that identifying both central and peripheral individuals may offer new insights for research on social representations.

## 1 Introduction

Recently, it has been proposed to study social representations (SRs) as networks with nodes representing not only individuals but also the opinions they express (Moliner 2023; Rizzoli et al. 2025). This approach differs from traditional models, which consider only networks of opinions, and offers the advantage of identifying distinct communities within the studied populations (Moliner 2023). In other words, it enables verification that the surveyed sample forms a homogeneous group with respect to the SR object under study. Additionally, this approach opens up new possibilities for SR research. Specifically, modeling an SR as a network that includes both opinions and individuals allows us to apply network metrics to individuals. Several indicators derived from these metrics pertain to node centrality, which makes it possible, in principle, to identify individuals who are central within an SR network in a given population. This research represents a preliminary exploration of this possibility.

## 2 Social Representations as Networks

We will not delve into the details of SR theory here (Moscovici 1961/1976) but will instead highlight specific aspects of representations that are relevant to our focus. Empirically, SRs are understood as sets of opinions or beliefs that members of a group associate with particular objects in their environment (Rateau et al. 2011). Within these sets, some elements enjoy relatively strong consensus among group members, while others represent minority viewpoints. Thus, in studying a stabilized SR—one whose emergence phase has concluded—we observe that the distribution of opinions and beliefs follows an exponential function. This distribution reflects the fact that, in a stabilized SR, only a few elements achieve broad consensus (Flament & Rouquette 2003; Moliner & Lo Monaco 2017).

Another important aspect of SRs is their close connection to communication processes. These processes are fundamental to both the emergence and evolution of SRs (Sammut et al. 2015). On one hand, these processes occur across different levels, from interpersonal communication to mass communication (Moliner 2001). On the other hand, depending on the context, they can have varied effects—from the social sharing of opinions and beliefs, which fosters consensus (Wagner & Hayes 2005), to polemics, which lead to the confrontation of differing SRs concerning the same object (Páez & Pérez 2020). This continuum includes normalization processes that help stabilize an SR, as well as influence processes that can drive its evolution (Castro et al. 2010).

Early SR studies focused on the structure of the various elements within these sets. The initial idea was that, within an SR, the stronger the connection between two elements, the greater the number of individuals who simultaneously express them (Flament 1981). Based on this principle, the technique of similarity analysis was developed (Degenne & Vergès 1973; Flament 1962). This approach creates a graphical representation of the structure of the set of opinions under study, in other words, a depiction that shows the elements of the SR and the primary links between these elements.

More recently, and following a similar principle, researchers have drawn on social network analysis to study SRs (Jung & Pawlowski 2014; Pawlowski et al. 2007; Pawlowski & Jung 2015). In this approach, different opinions or beliefs are considered the nodes of the network, while the links between each pair of nodes represent the number of individuals who simultaneously expressed or selected the opinions or beliefs associated with those nodes. By applying network metrics to such structures (Borgatti & Everett 2000; Freeman 1978), researchers can, for example, identify subsets of highly connected nodes that form the network core (Borgatti et al. 2002). However, since this approach does not incorporate individuals directly, it provides no information about the individuals themselves.

## 3 Network Analysis of Social Representations

**Figure 1:**
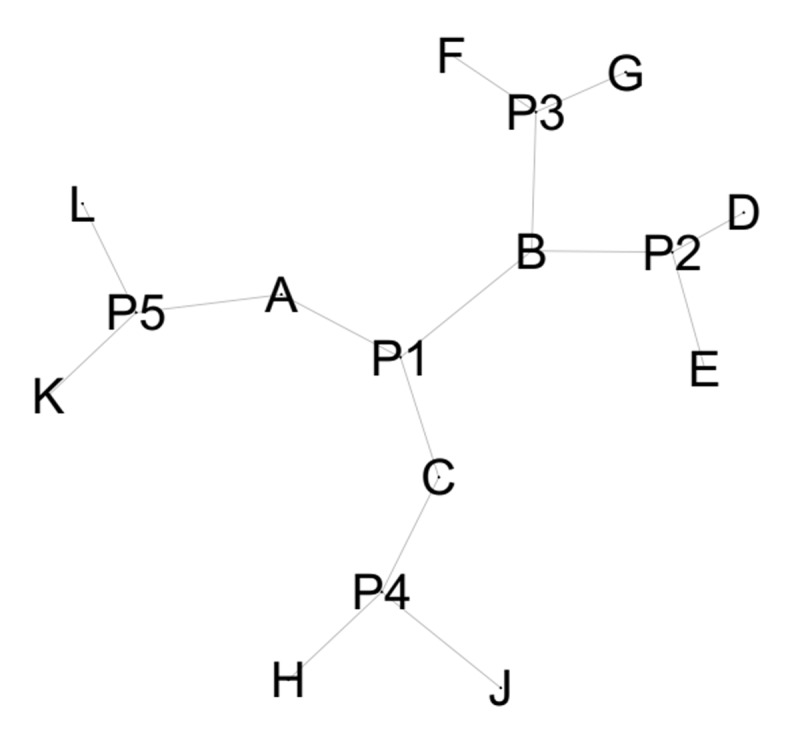
Network representation of the data in Table 1. Note: All figures in this paper showing networks, and all calculations on these networks, have been performed using Gephi 0.10 software.

To advance beyond the approaches described above, it has been proposed to analyze SRs as networks that include both opinions and individuals (Moliner 2023). In this technique, each node in the network represents either an opinion or an individual, and each node representing an individual is linked to nodes representing the opinions that individual expressed or selected. For example, if five participants are each asked to produce three verbal associations, the resulting data (Table 1) would be represented as shown in Figure 1.

**Table 1:** Five participants (P) produce three verbal associations (VA) each.


	VA1	VA2	VA3

P1	A	B	C

P2	B	D	E

P3	F	G	B

P4	J	C	H

P5	A	K	L


This approach yields a bipartite network (Borgatti & Everett 1997), meaning a network in which nodes are divided into two distinct subsets (individuals and verbal associations), and links are only possible between nodes belonging to different subsets. This method is part of a longstanding tradition of quantitative studies on social representations that allow for the visualization of representational structures (Degenne & Vergès 1973; Doise et al. 2014; Flament 1981; Pawlowski & Jung 2015). At the same time, it bears strong similarities to research on consensus formation and opinion dynamics in social networks (e.g., Ahmed et al. 2020; Kan et al. 2023; Rehman et al. 2023). Moreover, the proposed approach can be applied to various types of data. For instance, it may be adapted to questionnaire data, in line with methods used in network-based studies of attitudes and beliefs (e.g. Brandt et al. 2019; Dinkelberg et al. 2021; Leifeld 2020). For example, Carpentras and colleagues (2024) suggest treating each possible response option to a questionnaire item as a distinct node in the network. Thus, an item such as ‘Vaccines are useful,’ with five response options (from 1 = strongly disagree to 5 = strongly agree), would be represented by five separate nodes within the network.

It is therefore clear that the network-based approach we propose for the analysis of SR is closely related to existing research on the analysis of opinions, attitudes or beliefs networks. However, most of these studies consider networks in which the nodes represent either opinions or individuals but rarely incorporate both types of nodes simultaneously. Furthermore, in cases where both individuals and opinions are included (e.g., Carpentras et al. 2024; Dinkelberg et al. 2021; Leifeld 2020), these studies are generally not situated within the theoretical framework of social representations.

## 4 Network Metrics

Network researchers (Bavelas 1950; Beauchamp 1965; Freeman 1978) have long sought to characterize the nodes of a network using graph theory. As a result, several centrality measures have been developed (see e.g., Lee 2006; Li et al. 2015; Oldham et al. 2019; Valente et al. 2008). While we cannot cover them all here, we will focus on the one used in our research: eigenvector centrality.

Eigenvector centrality incorporates the concept of connectedness. The more connected a node is to other nodes that also have many connections, the more central it becomes. For example, in Figure 1, node P1 is the most central because all the nodes to which it is connected (A, B, C) are, themselves, connected to several other nodes. In the context of studying SRs, we must consider the meaning of this centrality measure when applied to individuals within a projection (see below) of the bipartite network of people holding opinions. Since the most commonly held opinions are the most connected, an individual will have higher eigenvector centrality in this type of network if they have chosen or expressed the most frequent opinions in the network. In this way, he is connected to nodes representing opinions, which in turn are connected to many other individuals. Ultimately, when applied to individuals in the context of SRs or collective opinion studies, eigenvector centrality reflects the intra-group frequency of the opinions chosen or expressed by those individuals. Thus, the most central individuals in a group are those who hold the most shared opinions within that group, making them the most typical members.

It is important to note that centrality measures—including eigenvector centrality—are based on algorithms originally developed for one-mode networks, that is, networks composed of nodes of the same type. However, the networks we propose to analyze are two-mode (bipartite) networks, as previously described. As a result, strictly speaking, applying the eigenvector centrality algorithm directly to a two-mode network can yield inaccurate or misleading results. To address this issue, researchers have introduced a ‘projection’ method that transforms a two-mode network into two distinct one-mode networks (Wilson 1982). The underlying principle is relatively straightforward: within an individual/opinion network, two individuals can be considered connected if they have selected at least one identical opinion. The strength of the tie between them increases with the number of shared opinions. Similarly, two opinions are considered connected if they have been selected by the same individual, with the tie’s strength increasing based on how many individuals selected both opinions. On this basis, a two-mode individual/opinion network can be decomposed into two one-mode networks: one representing the relationships between individuals, and the other representing associations between opinions. While this projection inevitably involves some loss of information, this loss is considered relatively minor (Borgatti & Everett 1997), and the method offers the significant advantage of ensuring the proper application of centrality algorithms.

## 5 Do central Individuals Have Specific Characteristics?

If it seems possible to identify central individuals in an SR network, the question then arises about their specificity. In other words, is occupying a central position associated with particular attributes or behaviors? This question has already been addressed in the context of networks where individuals are in physical contact (e.g., Fang et al. 2015; Jung & Rho 2020; Reinholt et al. 2011). However, it has not been explored in the context of the networks we propose to study. Yet, with regard to the interpersonal interactions that accompany the SR phenomenon (Moscovici 1961/1976), eigenvector centrality warrants attention. As we’ve seen, this measure reflects the typicality of individuals. This typicality can be explained in at least two ways. It could be the result of a mere artifact, where certain individuals happen to express majority opinions. Alternatively, it could reflect the ability of some individuals to accurately perceive and align with the consensus within their group.

We might also wonder whether the centrality of individuals is associated with a greater ability or willingness to engage in discussions with others about the object of representation. Indeed, one might argue that adhering to majority or widely held opinions within a group would protect individuals from potential criticism in such discussions. In other words, individuals who are more typical of a given group would likely encounter fewer conflicts in their interactions with other group members. Finally, we might ask what role central individuals play in stabilizing SRs. By holding majority positions, they could help increase the visibility of these opinions, making them more salient and perhaps more difficult to challenge.

For now, the possibilities outlined above are merely speculations that are difficult to translate into testable hypotheses. After all, we are dealing with virtual networks, not physical ones. At this point, we know very little about the properties of these networks, so it would be challenging to base any concrete hypotheses on them. This explains the exploratory nature of the research presented here.

## 6 An Exploratory Study: American Republican Voters and Climate Change

Building on the speculations just mentioned, we chose to use a dataset collected from recent research on climate change (Dixon et al. 2024). This choice was justified for at least two reasons. First, climate change serves as an ideal SR object, as it is a phenomenon that raises many questions and uncertainties within the population (e.g., Dézma et al. 2024; Jaspal et al. 2014; Mambet Doue et al. 2020). Second, it seemed to us that many of the questions posed to participants in this research overlapped in a relevant way with several aspects of our own problem. The dataset we used can be accessed via the following link: https://osf.io/zvk7r/?view_only=45974e96dbdb4a099ceffd10ae16034f.

### 6.1 Methods

#### 6.1.1 Participants

The study population consisted of a representative sample of 1,000 US Republican Party voters (*M*age = 53.5, *SD* = 17, 52.1% female). We did not have any other demographic information on the participants. The sampling and survey were carried out by the YouGov Institute from December 7, 2022, to December 14, 2022. Participants were surveyed using an online questionnaire.

#### 6.1.2 Questionnaire

The questionnaire contained two sets of questions (see online supplementary materials Appendix). The first set concerned participants’ support for five climate change mitigation policies and estimations of the percentage of Republican Party voters who support each of these five policies.

A second set of questions focused on participants’ social interactions about the 5 climate change mitigation policies.

- Here, four questions focused on participants’ past interactions about renewable energy and climate change adaptation policies, either on social networks or face to face.
- Two other questions focused on participants’ willingness to share their opinions with other Republican Party voters (one question about renewable energy and one about climate change adaptation policies).
- Six questions were designed to assess the extent to which participants anticipated conflicting interactions with other Republican Party voters when discussing climate change.
- Five questions focused on participants’ perceptions of the direction of information available in their environment (from social media, television, the press, colleagues, friends, or family). The question was whether participants thought this information portrayed Republicans as favorable to the five climate change policies.

#### 6.1.3 Data preprocessing

In our pre-processing of the responses to some of these questions, the original data were inverted because the coding of these responses went against the direction of the scales suggested to the participants. Responses corresponding to the consensus estimates were retained in their original form.

The five questions relating to support for climate change adaptation policies (1 = strongly opposed, 6 = strongly support, Cronbach’s alpha = .89) were aggregated into a single CCS index (Climate Change Sensibility = mean of the five responses).

The four questions on the frequency of past interactions (1 = never to 5 = almost always, Cronbach’s alpha = .87) were aggregated into a single POS index (Past Opinion Sharing = average of the four responses).

The two questions on willingness to share opinions (1 = very unwilling to 6 = very willing, Cronbach’s alpha = .93) were combined into a single WOS index (Willingness to Share Opinions = mean of the two responses).

The six questions on anticipation of conflictual interactions (Cronbach’s alpha = .96) were combined into a single ACI index (Anticipation of Conflictual Interactions = mean of the six responses). This index ranges from 1 to 6, with 6 indicating maximum anticipation of conflictual interactions.

The five questions on participants’ assessment of the direction of the information available in their environment (Cronbach’s alpha = .89) were aggregated into a single IEP index (Information Environment Perceptions = mean of the five responses).

### 6.2 Results

#### 6.2.1 Network

**Figure 2:**
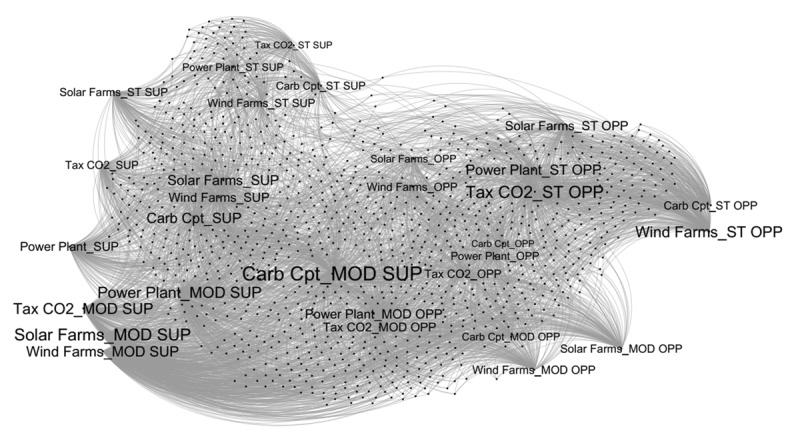
Network of 30 opinions and 1,000 participants on climate change mitigation policies (1,030 nodes and 5,000 links). Note. ST SUP = Strongly support, SUP = Support, MOD SUP = Moderate support, MOD OP = Moderate oppose, OP = Oppose, ST OP = Strongly opposed.

The first step in processing the collected data was to construct a network of opinions and survey participants. To do this, following a procedure adopted by Carpentras et al. (2024), we treated the responses to the approval scales for the five policies presented to the participants as distinct opinions. This resulted in 30 different opinions—5 policies combined with 6 types of responses (1 = strongly opposed, 2 = oppose, 3 = moderate oppose, 4 = moderate support, 5 = support, 6 = strongly support). Figure 2 shows the network derived from these 30 opinions.

**Figure 3:**
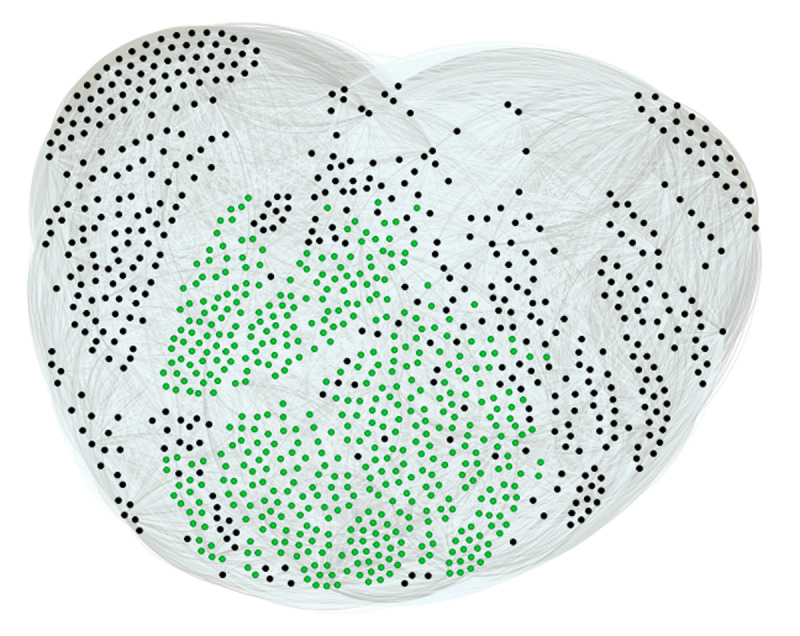
Network of the participants to the climate change mitigation policies survey (1,000 nodes and 269,565 links). Note: The network was built with the MultiMode Networks Projection plugin, Gephi 0.10. Green dots represent participants whose eigenvector centrality score is => at the median score. Links between participants have weights ranging from 1 (a single identical response) to 5 (5 identical responses).

After projection (see above) of this network, we obtain the network shown in Figure 3, which perfectly fits our problem, since the more a participant has chosen majority answers, the more he is connected to other participants and the more central he is. We therefore used the network in Figure 3 to calculate an eigenvector centrality score for each participant (*M* = 0.689, *Mdn* = 0.739, *SD* = 0.171, 95% CI [0.688, 0.709]). The Shapiro-Wilk W test indicates an abnormal distribution of this score (W = 0.94, p < .001).

To best assess the effects of participants’ centrality, we divided them into two groups according to their median centrality score (Centrality Low < 0.739, *N* = 500; Centrality High => 0.739, *N* = 500). Note that these numbers are well above what is recommended for detecting differences in means (two-tailed t-test) with a weak to moderate effect and a power of .80 (Cohen’s d = .30, p = .05, power = .80: *N* = 176). Table 2 shows the mean values and comparisons of the CCS, POS, WOS, ACI, and IEP scores. Because the distributions of all these scores are non-normal (Shapiro–Wilk W test, p < .001 for all scores), comparisons were performed using the Mann–Whitney U test.

**Table 2:** Mean scores, CCS, POS, WOS, ACI and IEP in central and peripheral participants.


	CENTRALITY	M	95% CI	SD	MDN	U	p	EFFECT SIZE

CCS	High	3.48	3.41, 3.56	0.82	3.6	119791	0.25	

	Low	3.35	3.20, 3.51	1.73	3.2			

POS	High	2.51	2.41, 2.60	1.04	2.5	108296	<.001	0.11

	Low	2.81	2.70, 2.91	1.21	2.75			

WOS	High	4.66	4.57, 4.76	1.03	5	104501	<.001	0.14

	Low	4.88	4.78, 4.99	1.2	5			

ACI	High	2.19	2.10, 2.28	1.02	2	132470	0.09	

	Low	2.15	2.05, 2.25	1.16	2			

IEP	High	3.48	3.40, 3.55	0.84	3.4	134530	0.03	0.06

	Low	3.33	3.21, 3.45	1.37	3.2			


Medians and comparisons (Mann-Whitney U test). CCS = Climate Change Sensibility, POS = Past Opinion Sharing, WOS = Willingness to Share Opinions, ACI = Anticipation of Conflictual Interactions, IEP = Information Environment Perceptions.

#### 6.2.2 Centrality and opinions

For the CCS score, no difference was observed between central and peripheral participants. Support for climate change adaptation policies is therefore identical in both groups.

#### 6.2.3 Centrality and social interactions

As can be seen (Table 2), central participants significantly differ from peripheral participants in their past interactions (POS) and their willingness to share opinions (WOS). They report fewer past interactions and express less willingness to share their opinions. However, the observed effects sizes are small. What’s more, this finding seems difficult to explain on the grounds that central participants would anticipate more conflictual interactions by sharing their opinions. In fact, there was no difference between the two subgroups of participants in terms of the average conflict anticipation score (ACI). On the other hand, we find that central participants are more likely than peripheral participants to believe that the information available in their environment shows that Republicans are favorable to climate change adaptation policies (IEP). However, the size of the effect found suggests that this difference is negligible. But when we examine the correlation between the information environment perception score (IEP score) and support for the five policies presented (CCS score), we find that it is both significant and high (Rho(998) = 0.74, p < .001). So, everyone is adjusting their opinions to what they perceive in their information environment.

To ensure that the observed results were not simply an artifact of the eigenvector centrality calculation, we ran a Monte Carlo simulation (1,000 iterations),^1^ building a new random network each time. Like the network shown in Figure 3, each simulated network consisted of 1,000 nodes and 269,565 links. At each iteration, the nodes of the simulated network were divided into two subgroups according to the value of their eigenvector centrality score (< median vs => median). Finally, at each iteration, the POS, WOS, ACI, and IEP scores were compared between the two subgroups using the Mann-Whitney U test. For the POS score, over the 1,000 simulations, the probability of obtaining the U value observed in Table 2 is equal to .001, for the WOS score it is less than .001. For the ACI score, the probability is .08 and for the IEP score, .03. We can therefore conclude that the differences observed in Table 2 for the POS and WOS scores, are probably not the result of a bias related to the calculation of the centrality score.

#### 6.2.4 Centrality and accuracy of consensus perception

**Figure 4:**
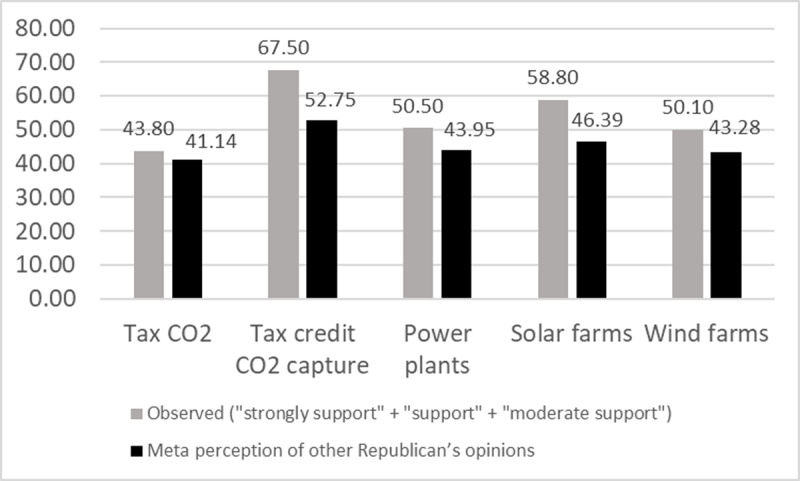
Observed (‘strongly support’ + ‘support’ + ‘moderate support’) and estimated support for the five policies presented in the questionnaire. Note. Observed: To what extent do you support or oppose the following proposals? Meta perception of other Republican’s opinions: What percept of Republican voters do you think support the following proposals?

For the 1,000 respondents, Figure 4 shows the proportion of participants who support each of the five policies presented in the questionnaire. It also displays the participants’ average estimates of support for these policies among Republican voters.

It can be seen that, overall, these estimates are systematically lower than the support observed among the 1,000 participants, with the exception of the policy of taxing CO_2_ emissions (two proportions z-test, taxing CO_2_: z = 1.20, p = 0.11; tax credit CO_2_: z = 6.73, p < .001; restrictions on power plants: z = 2.93, p < .01; solar farms: z = 5.55, p < .001; wind farms: z = 3.05, p = .0011). This phenomenon is obviously reminiscent of a pluralistic ignorance effect (Prentice & Miller 1993; Sargent & Newman 2021), whereby individuals may share an opinion in large numbers but remain ignorant of the consensual nature of that opinion. Several papers have highlighted this in the context of climate change (Geiger & Swim 2016; Kjeldahl & Hendricks 2018). But are all participants in our study subject to this effect? To answer this question, we calculated a Consensus Perception Accuracy score (CPA).

To determine this score for each participant, we calculated for each policy the absolute difference between the proportion of ‘strongly support,’ ‘support,’ and ‘moderate support’ responses observed in our population and the participant’s estimate. We then averaged these differences for each participant. For example, a participant who estimated that 100% of Republican voters support each policy would receive an average of 45.86 (ABS(100–43.80) + ABS(100–67.50) + ABS(100–50.50) + ABS(100–58.80) + ABS(100–50.10) = 229.30 / 5 = 45.86). To obtain a score ranging from 0 to 1, this participant’s score is (100–45.86) / 100 = 0.54. Following this approach, we assigned each participant a CPA score, with a score of 1 indicating that the participant made a perfectly accurate estimate of the consensus observed in the group (*M* = 0.75, *SD* = 0.12, 95% CI [0.74, 0.76]). The Shapiro-Wilk W test indicates an abnormal distribution of this score (W = 0.97, p < .001).

We find that the mean CPA score is significantly higher in central participants than in peripheral participants (*M* = 0.79, *SD* = 0.10, 95% CI [0.78, 0.80], *Mdn* = 0.80 vs *M* = 0.71, *SD* = 0.13, 95% CI [0.70, 0.72], *Mdn* = 0.71, U = 169051, p < .001, effect size = 0.30), which indicates that central participants have a better perception of consensus within the group of Republican Party voters.

Again, we ran a Monte Carlo simulation on 1,000 random networks (1000 nodes and 269565 links). This simulation indicates that the probability of observing the U-value of 169051 for the CPA score comparison is less than .001.

#### 6.2.5 In Search of a Representative Central Minority

Given the small effect size of differences found between central and peripheral participants (see Table 2), we compared the minority of the most central participants to the rest of the group. Since central individuals adhere to the majority opinions of their group, it’s clear that their responses are a good approximation of the opinions of the group as a whole. But what about the other measures? Do they have the same frequency of social interaction? Do they have the same perceptions of their information environment? To answer these questions, we looked at two subgroups of participants. The first corresponds to the most central individuals (*N* = 250) and includes participants whose score is above the boundary of the last quartile of the centrality score distribution (0.83). The second corresponds to the remaining participants (*N* = 750). Table 3 shows the comparisons between the two subgroups.

**Table 3:** Mean scores, CCS, POS, WOS, ACI, CPA and IEP in central and other participants.


	GROUP	M	95% CI	SD	Mdn	U	p	EFFECT SIZE

CCS	Central	3.34	3.25, 3.44	0.76	3.50	88447	0.18	

	Other	3.44	3.34, 3.55	1.50	3.40			

POS	Central	2.56	2.44, 2.69	1.00	2.50	90177	0.38	

	Other	2.68	2.60, 2.77	1.18	2.50			

WOS	Central	4.70	4.57, 4.83	1.02	5.00	85480	0.03	0.06

	Other	4.79	4.71, 4.88	1.15	5.00			

ACI	Central	2.18	2.05, 2.32	1.06	2.00	91536	0.58	

	Other	2.16	2.09, 2.24	1.10	2.00			

CPA	Central	0.79	0.78, 0.80	0.11	0.81	117792	<.001	0.19

	Other	0.73	0.72, 0.74	0.12	0.75			

IEP	Central	3.35	3.25, 3.45	0.80	3.40	90580	0.44	

	Other	3.42	3.33, 3.51	1.23	3.40			


Medians and comparisons (Mann-Whitney U test). CCS = Climate Change Sensibility, POS = Past Opinion Sharing, WOS = Willingness to Share Opinions, ACI = Anticipation of Conflictual Interactions, IEP = Information Environment Perceptions.

We find that there are no significant differences between the most central participants and the others for 4 measures (CCS, POS, ACI, and IEP). Monte Carlo simulation results suggest that this lack of difference is not due to an artifact related to the calculation of centrality (CCS p < .001, POS p < .001, ACI p < .001, IEP p < .001). On the other hand, we find a significant difference for the WOS score, but with a very small effect size. Although Monte Carlo simulation suggests that this difference is not due to artifact (p < .001), it can be considered negligible. Finally, there is a significant difference for the CPA score, but the Monte Carlo simulation result does not confirm that this difference is not due to an artifact related to the centrality measure (p = 0.12). This set of results therefore leads us to conclude that the most central participants represent a minority that is fairly representative of the group as a whole. Apart from the fact that the members of this minority may have a better perception of the consensus within the group.

#### 6.2.6 In search of a peripheral minority

The questions just raised about the most central participants also apply to those who are most peripheral. Do they have the same frequency of social interaction? Do they have the same perceptions of their information environment? To answer these questions, we again considered two subgroups of participants. The first corresponds to the most peripheral individuals (*N* = 247) and includes participants whose score is less than the boundary of the first quartile of the centrality score distribution (0.55). The second corresponds to the remaining participants (*N* = 753). Table 4 shows the comparisons between the two groups.

**Table 4:** Mean scores, CCS, POS, WOS, ACI, CPA, and IEP in peripheral and other participants.


	GROUP	M	95% CI	SD	Mdn	U	p	EFFECT SIZE

CCS	Other	3.47	3.40, 3.54	0.99	3.60	87343	0.15	

	Periph	3.26	3.00, 3.53	2.10	2.40			

POS	Other	2.56	2.48, 2.64	1.10	2.50	76623	<.001	0.17

	Periph	2.93	2.79, 3.09	1.19	3.00			

WOS	Other	4.65	4.57, 4.73	1.11	5.00	65776	<.001	0.29

	Periph	5.13	5.00, 5.27	1.08	5.00			

ACI	Other	2.21	2.14, 2.29	1.06	2.00	79333	<.001	0.14

	Periph	2.02	1.88, 2.17	1.15	2.00			

CPA	Other	0.77	0.77, 0.78	0.11	0.79	51875	<.001	0.44

	Periph	0.67	0.65, 0.69	0.13	0.66			

IEP	Other	3.46	3.39, 3.53	0.95	3.40	82491	0.008	0.11

	Periph	3.22	3.03, 3.42	1.57	3.00			


Medians and comparisons (Mann-Whitney U test). CCS = Climate Change Sensibility, POS = Past Opinion Sharing, WOS = Willingness to Share Opinions, ACI = Anticipation of Conflictual Interactions, IEP = Information Environment Perceptions.

Although the two groups do not differ in their opinions on climate change adaptation policies (CCS), the peripheral minority participants are significantly more active than the others in terms of social interactions (POS and WOS). They expect significantly less conflictual interactions than others (ACI) and have a lower perception of consensus within the group (CPA). Finally, they are significantly less likely than others to believe that the information available in their environment suggests that Republicans are in favor of climate change adaptation policies (IEP). The Monte Carlo simulation results indicate that the U-values in Table 4 are unlikely to be due to artifact (p < .001), except for the WOS score (p = 0.29).

**Figure 5:**
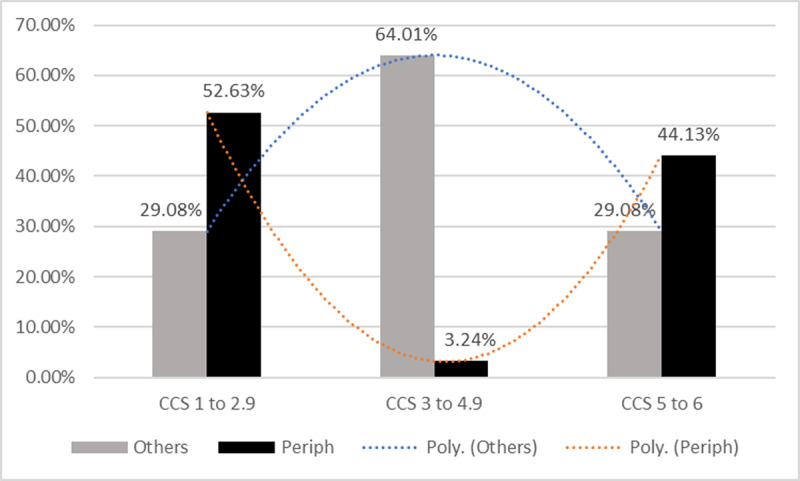
Percentage of participants from the peripheral minority and percentage of participants from the rest of the group (others) by level of support for climate change adaptation policies (CCS score).

These results may be surprising in that they suggest the presence of a minority (the most peripheral participants) who would be more opinion-sharing than the rest of the group (POS score). But why would they want to share their opinions if they’re more or less identical to those of the rest of the group (CCS score)? Figure 5 provides an answer to this question.

**Figure 6:**
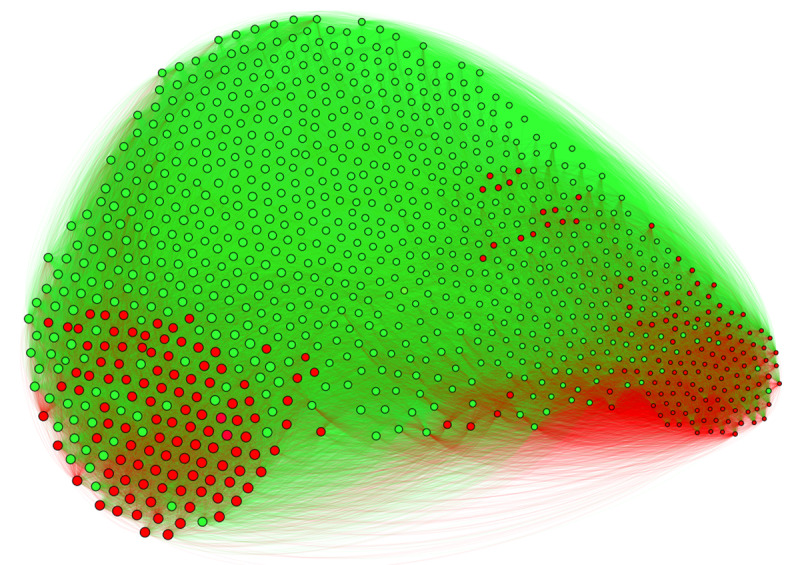
**Participant network projection (1000 nodes and 269565 links)**. Red: most peripheral participants (n = 247). Green: rest of the group. The size of the dots is proportional to the score of agreement to the five climate change adaptation policies (CCS score).

As can be seen, the minority of the 247 peripheral participants can be divided into two subgroups, one very unfavorable to adaptation policies and the other very favorable. On the other hand, in the rest of the group, almost two-thirds of the participants have moderate positions. These distributions explain why the overall CCS score of the peripheral minority does not differ from that of the rest of the group. Figure 6 shows the projected participants’ network, with the two peripheral minorities represented in red and the rest of the group in green.

The size of the dots representing the participants in this figure is proportional to their score of support for the five climate change adaptation policies (CCS score). Thus, on the left of the network are the participants who are most in favor of these policies (*N* = 113), and on the right are those who are least in favor (*N =* 134). The positioning of the most peripheral participants in this network suggests the existence of two very distinct peripheral communities.

The most interesting result here is the polarization phenomenon between these two communities, as suggested by Figure 6. This phenomenon is supported by the comparison of the CCS scores of the minority in favor of adaptation measures and the minority opposed to them (*M* = 5.47, 95% CI [5.40, 5.55], *SD* = 0.41 vs. *M* = 1.40, 95% CI [1.29, 1.50], *SD* = 0.60; U = 1000, p < .001, effect size = 0.99). We can therefore see that within each minority, participants’ positions tend to cluster toward the extremes of the response scale provided (1 = strongly opposed, 6 = strongly support).

## 7. Discussion

We have seen that in the network we studied; peripheral individuals report having had more past social interactions about climate change adaptation measures than central individuals. Similarly, they express a greater willingness than central individuals to engage in future interactions on these issues. In contrast, central individuals have a better perception of the consensus within the group of Republican voters and the most central of them represent a minority that is fairly representative of the group as a whole. Finally, we observed that the most peripheral individuals in this network do not form a homogeneous group: they are either strongly opposed to or strongly supportive of the adaptation measures presented to them. Conversely, the most central individuals in the network do not hold extreme opinions regarding these climate change adaptation measures.

We will not discuss here the implications of our results regarding the link between individuals and climate change. Our primary goal was to provide empirical evidence supporting the idea that identifying central and peripheral individuals in opinion networks may be valuable. We believe the study we have just presented moves in this direction. However, we must point out an important limitation of this work. In this study, we focused on five policies to combat global warming and their combinations with six levels of support or opposition. However, it is clear that the SR of climate change among American Republican Party voters is not limited to their support or opposition to the policies presented. This SR likely includes many other opinions, some of which may be far more important than those considered in our study. This means we have explored only one aspect of climate change SR within the surveyed population. It also means that the central individuals we identified are only central to the specific area of climate change SR explored in this work. With this in mind, there is no reason to believe that if we had a broader range of opinions, we might not have observed different results from those we have presented. Despite this limitation, which obviously reduces the scope of our findings, we believe our research raises at least four important questions worth discussing.

The first concerns the determinants of centrality. We may wonder why certain individuals hold opinions that are among the most common in the group to which they belong. We are obviously thinking of a classical conformity effect (Asch 1951), motivated by individual dispositions (e.g., gregariousness) or fear of being marginalized. But we could also invoke a well-known consequence of social comparison (Festinger 1954), which leads to in-group uniformity (Brehm & Festinger 1957). However, this explanation is contradicted by some of our results. As we have seen (see Table 3), the opinions of the most central individuals are majority opinions within the group. We can therefore assume that they have not experienced more social comparison situations in which they might have been under pressure to be uniform. Finally, there are factors related to the environment of the central individuals and their exposure to the media. Indeed, in terms of SR theory, we might think that an issue such as climate change still generates a great deal of uncertainty and questioning. We might then expect that, in this context, certain individuals would align their opinions with those they encounter most frequently, precisely in response to the uncertainty and questioning they face. We have seen that the IEP score of the central participants was significantly lower than that of the peripheral participants (see Table 2). The observed effect is negligible (.07), but it gives us a clue. Indeed, we found a strong correlation between CCS and IEP scores. This suggests that individuals adjust their opinions based on what they perceive in their informational environment. If these perceptions were accurate, i.e., if they correctly reflected the reality of each other’s information environments, we’d have an explanation for centrality. In other words, we could say that the information environment of central individuals presents them with majority opinions among Republican voters and moderately favorable to adaptation policies, while the information environment of peripheral individuals presents them with minority opinions among Republican voters. Of course, this explanation requires further research, and the question of centrality remains unanswered for now.

The second issue to be discussed is the more frequent social interactions of peripheral minority participants (see Table 4, POS score). The observed phenomenon corresponds quite well to what happens in ‘active minorities’ (Moscovici 1976; Moscovici 1979), whose members try to influence other group members through their social interactions. As shown in Figures 5 and 6, the minority of the 247 most peripheral participants is divided into two subgroups, one very unfavorable to adaptation policies and the other very favorable. According to minority influence theory, we can therefore expect that Republican Party voters, as a whole, will eventually adopt one of these two positions. Identifying peripheral individuals could therefore help to anticipate the likely evolution of a SR within a group.

The third question raised by our results concerns the accuracy of consensus perception. How can we explain the fact that some individuals perceive consensus within their group better than others? The phenomenon of social projection has long been recognized (Katz & Allport 1931). When it comes to evaluating the choices, preferences, or beliefs of others, we tend to project our own choices, preferences, or beliefs onto others. This tendency sometimes leads to false consensus effects (Ross et al. 1977), but in this case it may explain the more accurate perceptions of the central participants. Indeed, we saw that the population we surveyed was, on the whole, moderately supportive of climate change policies (see Figure 4). But we also saw that the position of central participants reflected the position of other participants quite well (see Table 3, CCS score). In other words, if central participants make better estimates of the group consensus, it may be because they are simply projecting their personal position in making these estimates.

The final question we need to address here concerns the relationship between individuals’ centrality within a SR network and the centrality of the elements (opinions or beliefs) of a SR, in the sense of core theory (Abric 1976; 1993). According to this theory, every stabilized SR is composed of a central system and a peripheral system.

The central system consists of a small number of elements that concentrate the meanings a group associates with an object in its environment. These elements are the subject of broad consensus within the group. For this reason, the central system contributes to the stability of the SR within the group.

The peripheral system, by contrast, includes a much larger number of elements than the central system. These elements correspond to individual experiences and reflect the diversity of those experiences. Consequently, they are the subject of weaker consensus within the group. Thus, the peripheral system allows for a certain degree of heterogeneity within the group while enabling potential evolution of the SR.

The connection between core theory and individuals’ centrality within a SR network is quite evident. Since the central elements of a SR are among the most consensual, individuals who adhere to them are naturally the most central members of the network. This observation is relatively straightforward, but it may have important implications for understanding the dynamics of SRs.

We have seen that central individuals perceive group consensus more accurately than others. If this finding were to be confirmed, it would support the idea that central individuals play a specific role in maintaining the stability of social representations. Indeed, they adhere more strongly than others to core opinions and beliefs. In a sense, they are also aware that many other group members share these beliefs. We can therefore hypothesize that central individuals in a SR network may contribute to the stability of the SR, for example by being less inclined than others to change their beliefs, particularly those belonging to the central system.

## 8. Conclusion

We have just seen that a network analysis of a SR and the measurement of individuals’ centrality make it possible to reveal communities, dynamics of opposition, and patterns of interaction that would have been difficult to uncover otherwise, particularly through multivariate analyses. At this stage of our research, it would be premature to claim that the results we have just presented allow us to draw definitive conclusions about the central or peripheral position of individuals in SR networks. In particular, it is quite possible that what we have observed is specific to the particular context of the present study, both in terms of population and theme. Thus, it will undoubtedly be necessary to replicate the studies with different groups, on different topics, and with different measures in order to draw robust conclusions about the characteristics of central and peripheral individuals. However, it seems to us that the results just presented are promising and could open new perspectives for the study of SR.

## Additional File

The additional file for this article can be found as follows:

10.5334/irsp.1018.s1 Supplementary materials. Questionnaire.

## Footnotes

This simulation was performed using the Jamovi software (2.3.28) and the Rj-Editor module.
