# Supplementary material for: Central and Peripheral People in a Social Representation Network
Source: Int Rev Soc Psychol. 2025 Dec 4;38:16. doi: 10.5334/irsp.1018 (PMC12679991; doi:10.5334/irsp.1018)
Supplement: Supplementary materials. — Questionnaire. [file irsp-38-1018-s1.pdf]

Questionnaire.

The codes before the questions (e.g. Q3-1) correspond to the column codes in the data file.

Questions set 1.

To what extent do you support or oppose the following proposals to reduce the effects of global climate change? (1= Strongly support, 2= Support, 3= Awesome support, 4= Awesome oppose, 5= Oppose, 6= Strongly oppose).

- Q3-1 : Taxing corporations based on the amount of carbon emissions they produce.
- Q3-2 : Providing a tax credit to encourage businesses to develop technology which captures and stores carbon emissions so they do not enter the atmosphere.
- Q3-3 : Tougher restrictions on power plant carbon emissions.

To what extent do you support or oppose EXPANDING each of the following sources of energy in our country ? ? (1= Strongly support, 2= Support, 3= Awesome support, 4= Awesome oppose, 5= Oppose, 6= Strongly oppose).

- Q4-1 : More solar panel “farms”.
- Q4-2 : More wind turbine “farms”.

What percent of Republican voters do you think **support** the following proposals to reduce the effects of global climate change? (0% to 100%)

- Q11-a : Taxing corporations based on the amount of carbon emissions they produce.
- Q11-b : Providing a tax credit to encourage businesses to develop technology which captures and stores carbon emissions so they do not enter the atmosphere.
- Q11-c : Tougher restrictions on power plant carbon emissions.

What percent of Republican voters do you think you **support** expanding each of the following sources of energy in our country. (0% to 100%)

- Q12-a : More solar panel “farms”
- Q12-b : More wind turbine “farms”

Questions set 2.

To what extent have you shared your views about renewable energy? (1= Almost always, 2= often, 3= sometimes, 4= seldom, 5= never)

- Q24-1 : Face to face
- Q24-2 : On social media (e.g., Facebook, Twitter, Instagram, etc.)

To what extent have you shared your views about climate change policies? (1= Almost always, 2= often, 3= sometimes, 4= seldom, 5= never)

- Q25-1 : Face to face
- Q25-2 : On social media (e.g., Facebook, Twitter, Instagram, etc.)

Q21-7 : To what extent are you willing to share your opinions about renewable energy with other Republican voters? (1= very willing, 6= very unwilling).

Q21-8 : To what extent are you willing to share your opinions about climate change policies with other Republican voters? (1= very willing, 6= very unwilling).

Q27-7 : It is likely I will experience unpleasant discussions if I share my opinions about solar panel farms to other Republicans. (1 = strongly agree to 6 = strongly disagree)

Q27-8 : Conflict is likely if I discuss my position on alternative energy, like wind farms, with other Republicans.

Q27-9 : I'm worried that arguments will happen if I talk to other Republicans about my views on alternative energy.

Q27-10 : It is likely I will experience unpleasant discussions if I share my opinions carbon taxation to other Republicans.

Q27-11 : Conflict is likely if I discuss my position on climate change policies, like tax credits for carbon capture technology, with other Republicans.

Q27-12 : I'm worried that arguments will happen if I talk to other Republicans about my views on proposals to restrict carbon emissions in power plants.

Think about the environment in which you obtain information (e.g., social media, television, news media, friends, family, colleagues, etc.). To what extent do you agree or disagree with the following statements? (1= strongly agree, 6= strongly disagree)

Information about alternative energy that I've come across suggests that Republicans:
Q17-a : expanding solar panel "farms" as sources of energy in our country.
Q17-b : expanding wind turbine "farms" as sources of energy in our country.

Information about climate change policies that I've come across suggests that Republicans:
Q18-a : Taxing corporations based on the amount of carbon emissions they produce
Q18-b : Providing a tax credit to encourage businesses to develop technology which captures and stores carbon emissions so they do not enter the atmosphere.
Q18-c : Tougher restrictions on power plant carbon emissions.
